# Risk of disability among adult leprosy cases and determinants of delay in diagnosis in five states of India: A case-control study

**DOI:** 10.1371/journal.pntd.0007495

**Published:** 2019-06-27

**Authors:** Govindarajulu Srinivas, Thirumugam Muthuvel, Vivek Lal, Kanagasabapathy Vaikundanathan, Eva-Maria Schwienhorst-Stich, Christa Kasang

**Affiliations:** 1 Professor & Head, Department of Epidemiology, The Tamil Nadu Dr. M.G.R. Medical University, Chennai, India; 2 Epidemiologist, Global Data Research Center, Hyderabad, India; 3 Director-Health, German Leprosy and TB Relief Association, Kolkata, India; 4 Rehabilitation Officer, Composite Regional Centre for Persons with Disabilities, Davanagere, India; 5 Deutsche Lepra- und Tuberkulosehilfe e.V (DAHW) and Faculty of Medicine, University of Würzburg, Germany; 6 Research Consultant, Deutsche Lepra- und Tuberkulosehilfe e.V., Wuerzburg (DAHW), Germany; Kwame Nkrumah University of Science and Technology, GHANA

## Abstract

**Introduction:**

A high proportion of grade 2 disability (visible deformity) is indicative of delay in detection of leprosy and leprosy is one of the major causes of preventable disability. We conducted this study to determine the risk factors associated with disability (G2D and G1D) among adult new leprosy cases and to measure their strength of association.

**Methods:**

A multi-centric case-control study was undertaken in five states of India i.e. Andhra Pradesh, Delhi, Gujarat, Maharashtra and West Bengal). Among new adult patients, cases were defined as those with disability (G2D and G1D) at the time of diagnosis and controls were defined as those without any disability (G0D). Delays were quantified based on patient recall across a timeline. Patient delay defined as the time period between first noticed symptom by the patient and the first visit to any health care provider (HCP); HCP delay defined as the time period between patient’s first visit to any HCP and the confirmation of diagnosis of leprosy; and total delay defined as the sum of both patient and HCP delays.

**Results:**

A total of 1400 new leprosy patients (700 G2D/G1D and 700 G0D) across five states were interviewed. Among G2D/G1D, the median patient delay was 8 months compared with 4 months among G0D. The median HCP delay was 2 months for G2D/G1D and 1 month for G0D. The median total delay was 14 months for G2D/G1D and 6.2 months for G0D; observed median difference between groups was statistically significant (p<0.001). When patient delay was more than 3 months, odds of G2D/G1D at diagnosis were 1.6 times higher compared to when patient delay was less than 3 months. When the HCP delay was more than one month, the odds of G2D/G1D were 1.4 times higher compared to when the HCP delay was less than one month. When the patient had multi-bacillary type leprosy the odds of G2D/G1D at the time of diagnosis was nine times higher compared to pauci-bacillary type leprosy.

**Conclusion:**

Patient delay is the major reason for risk of disability (G2D/G1D) among adult leprosy patients. A patient delay of more than 3 months from the notice of first symptom is a significant indicator for the disabilities among adult leprosy patients. Early case detection campaigns like active surveys in endemic spots should be done periodically as this can reduce delays and promote early diagnosis. Additionally, the program should lay greater emphasis on raising community awareness regarding the disease. Also, health care provider delay of more than 1 month have been significant risk factors for disability among adult leprosy cases. Hence, periodical capacitation of all HCPs including private practitioners would significantly contribute to reduce diagnostic delay and promote timely referral and early detection.

## Author summary

Leprosy is an infectious disease caused by the *Mycobacterium leprae* and is one of the major causes of preventable disability. In the recent years there has been an increase in the number of new leprosy patients with disability in India. People affected by leprosy often experience severe stigmatization because of its disabling consequences. This neglected tropical disease continues to pose a major disease burden in India. Despite the availability of health facilities, there continues to be barriers towards leprosy diagnosis and early treatment. Assessment of risk factors for disability is important in the era of leprosy integration with general health services. Risk factors for disability related to the patient and health care provider were identified to help the policy makers to develop appropriate strategies to promote early diagnosis and prevent disability.

## Introduction

Leprosy is an infectious disease caused by the *Mycobacterium leprae and* is one of the important causes of preventable disability [1]. Early diagnosis and prompt treatment of all new cases of leprosy with World Health Organization (WHO) recommended multidrug therapy (MDT) remain the key strategies for leprosy control as it would prevent nerve damage and disability [2]. An early diagnosis also provides opportunities for reducing or halting further transmission. Despite that, there are many current reports across the world, showing that people are still diagnosed late for leprosy [3–5].

After the declaration of elimination of leprosy as a public health problem in India in 2006, leprosy services were integrated within the general health care system for early diagnosis, prompt treatment, and care [6]. Despite increased efforts, reports and studies suggest leprosy is diagnosed late [7–9]. India is one of the countries with the highest leprosy burden with more than 135,000 new leprosy patients being detected every year, including 5,245 (3.9%) new leprosy patients with a visible disability: grade 2 disability (G2D) [10]. In the year 2016, India reported 63% of the world’s new leprosy cases; about 40% of the world’s new G2D among new leprosy cases. India reported an increasing trend of new cases with G2D in the period 2008–2015 from 3.1% to 4.6% [11]. A total of 3,848 new cases with G2D was detected by contact examination in the leprosy case detection campaign in April 2017 to March 2018 [12]. Global Leprosy Strategy 2016–20, has set a goal to reduce leprosy burden assessed through a target of <1/million rate of newly diagnosed leprosy patients with visible deformities [6]. The number of G2D patients among new leprosy cases in 5 Indian States (Andhra Pradesh, Maharashtra, Gujarat, Delhi and West Bengal) was 1,913 (36% of India) and 2,067 (36% of India) in 2013–14 and 2014–15 respectively [13,14]. The number of cases with G2D at the time of diagnosis directly reflects the delay in the early detection; the level of leprosy awareness in the community; the capacity of the health system to recognize leprosy early and to some extent the reach of services [12].

Leprosy presents itself as a public health problem due to the disabilities it causes and the costs associated with its management [15]. Although several factors associated with the development of disabilities are addressed in the literature. However, studying the extent of each factor—alone or associated—empowers managers, health professionals and researchers in the implementation of preventive and curative strategies and would assist the leprosy programmers to prioritise the interventions [16]. The delayed presentation is a recognised risk factor for disability in leprosy and is the result of complex interactions between physical, social, economic and psychological factors [3,17]. Assessing the potential factors associated with delays in seeking care and diagnosis of leprosy is essential to identify program impediments and devise appropriate strategies to promote early diagnosis and prevent disability. The present study was conducted to determine the risk factors associated with disability (G2D and G1D) among adult new leprosy cases and to measure their strength of association. We hypothesized that adult leprosy cases who have a delay in diagnosis are at a greater risk of developing disability (G2D and G1D). In the present study, we quantified the delay in terms of patient delay and health care provider delay.

## Materials and methods

### Ethical considerations

Ethical approval was obtained from the Institutional Review Board (IRB) of German Leprosy and TB Relief Association (GLRA), India. Informed written consent was obtained from participants. Permission was taken from relevant authorities (State Leprosy Officers, Directorate of Health Services and District Leprosy Officers) before conduction of the study. Direct interviews were conducted with the study participants in health facilities by trained research assistants using a structured questionnaire after obtaining their informed consent. To maintain the confidentiality of the study participant’s data, a delinked number was assigned to each study participant. All the filled in written questionnaire was kept in a closed cabinet and will be preserved for a period of five years in GLRA, India office.

### Study design, period and area

**Fig 1:**
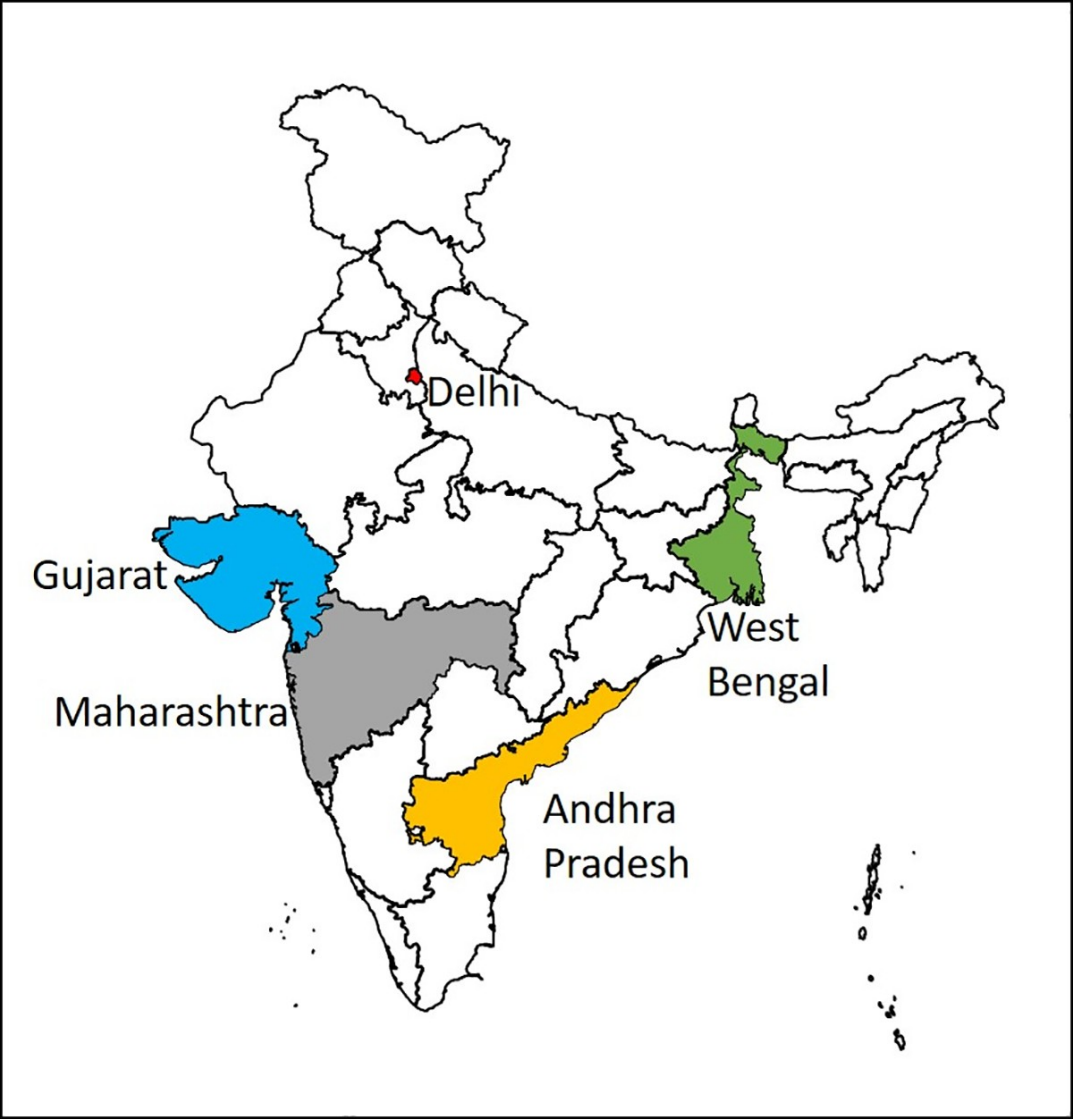
Geographic representation of data collection states in India.

A case-control study was undertaken during August 2014 to July 2016 (entire study duration) in five states in India, Andhra Pradesh, Maharashtra, Gujarat, Delhi and West Bengal (Fig 1). These five states were chosen based on the feasibility and leprosy burden reported in the preceding years of data collection.

### Source and study population

The study population were adult leprosy patients (18 years and older based on standard definition of adulthood) at the time of first diagnosis and registered for treatment under the National Leprosy Eradication Programme (NLEP). Cases and control group were identified and selected through the treatment registry, registered after 1^st^ January 2013 (in order to obtain the required sample size while at the same time attempting to reduce the recall bias). Cases were defined as leprosy cases with G2D or G1D, controls were without disability, meaning grade 0 disability (G0D), at the time of first diagnosis [18]. WHO G1D was defined as the patient developing anaesthesia in the palm or sole tested with a ballpoint pen and leprosy eye involvement with preservation of visual acuity better than 6/60 in conformity. G2D was defined as a visible deformity in either the hands, feet or eyes, both according to the WHO disability grading [18].

### Sample size

For each state the sample size required was calculated using Epi Info version 3.03.17; based on probability of exposure to one of the main risk factors (poor knowledge/low awareness of disease) as 20% in the control group and an expected odds ratio of 2, with power of 80% and an alpha error of 5% for one sided test, the minimum sample size required was 135 cases. Hence, for each state it was decided to select 140 cases and 140 controls. Thus, the total sample size for this study involving five states was 700 cases with disability (G2D or G1D) and 700 controls without disability (G0D).

### Sampling & selection of cases and controls

In order to select a representative sample of each state, a two-stage cluster sampling procedure was followed. The district was the primary sampling unit, and cases/ controls were the secondary sampling unit. The primary sampling unit (PSU) was selected through probability proportion to size (PPS). For each of the selected districts, the secondary sampling unit was a list of G1D and G2D leprosy cases in the order of registration date from the treatment registry. The required number of samples was selected randomly from the list by multistage random sampling. In a situation of not getting a participant (example: not getting consent for participation or non-availability of the selected participant), the next available case or control in the treatment register was enrolled into the study. Controls were selected in the respective blocks (sub-district level) of each district where the cases were recruited. Wherever the controls were not available, the next available control based on the leprosy register was approached and recruited.

### Data collection and quality control

Actual period of data collection was during the period January 2015 and January 2016 using a close-ended structured questionnaire by trained research staffs. The study tool and informed consent form were translated and back-translated into local languages of each of the five states (Hindi, Marathi, Gujarati, Bengali, and Telugu). The questionnaire was pre-tested on 5% of study participants before the actual data collection. The principal investigator supervised every aspect of the data collection to ensure data collected was error-free. The variables collected were educational status, occupation, place of residence, knowledge & awareness of the disease, quantification of patient delay, reasons for the patient delay, first health care provider (HCP) met, pathways of health care sought and quantification of HCP delays, etc.

### Minimizing recall bias

Delay in diagnosis was based upon the patient’s recall; as it might be subject to recall bias, the research assistants used months as the measuring unit. Interviewer spent about 40–50 minutes for each interview and recorded the responses (in written questionnaire) according to the necessity of each patient. A calendar was offered and provided to the patients, in case this was needed. Also, local or national events or religious festivals were referred for recall. Information on the WHO grading was collected from the existing treatment records and cross-checked with the program staffs whenever it needed clarity.

### Operational definitions of diagnostic delay

**Fig 2:**
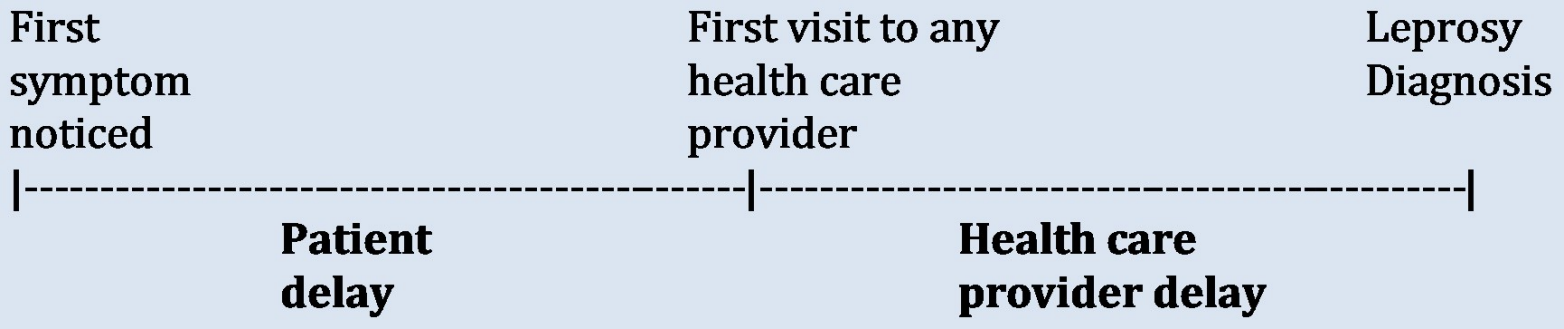
Types of diagnostic delay.

- **Patient Delay:** The period between the first noticed symptom by the patient and the first visit to any health care provider.
- **Health Care Provider (HCP) delay**: The period between the patient’s first visit to any health care provider and confirmation of the diagnosis of leprosy.
- **Total Diagnostic Delay:** The sum of both the patient delay and health care provider delay (Fig 2).

**Cases:** Cases were adult leprosy patients with G1D or G2D [as categorized by (WHO] at the time of first diagnosis and who were registered for treatment under the leprosy programme after 1^st^ Jan 2013 (to reduce recall bias).

**Controls:** Controls were adult leprosy patients with grade 0 disability [as categorized by WHO] at the time of first diagnosis and who were registered for treatment under the leprosy programme after 1^st^ Jan 2013 (to reduce recall bias).

### Statistical Analysis

Data was coded and then entered into EpiData entry software. The de-identified data from all project sites were merged into a centralized EpiData manager database, maintained at the GLRA head office, Chennai. The data were analysed using STATA version 12. Basic descriptive analyses were done. Mean (SD) or median inter-quartile range (IQR) was used to describe diagnosis delay. The median difference between cases and controls were analysed using the Mann-Whitney U test. Mann-Whitney U test was used because the delay (in months) was not normally distributed. The association between risk factors and disability was inferred using adjusted odds ratios (adj OR) and 95% confidence interval (95% CI). Univariate logistic regression was first fitted and those independent variables which become significant in the univariate regression at p-value of less than 0.05 were included in the multivariate logistic regression analysis. Backward stepwise multiple logistic regression was fitted to determine the net effect of each independent variable on late diagnosis of leprosy; using 5% level of significance.

## Results

### Participant characteristics

A total of 1,400 leprosy patients were enrolled across five states of India in this study, which included 700 cases and 700 controls. In each of the five states, 140 cases (70 patients with G2D or 70 patients with G1D) and 140 controls (G0D were interviewed. The participant’s socio-demographic characteristics were described in Table 1.

**Table 1:** General characteristics of G2D/G1D (cases) and G0D (controls) studied in the 5 states of India during 2014–2016.

Variables		G2D/G1D (n = 700)		G0D (n = 700)	
		n	(%)	n	(%)
**Patient’s Gender**					
Men		477	(68)	494	(71)
Women		223	(32)	206	(29)
**Age at diagnosis (Mean±SD) in years**		40.0±14.3		37.7±13.4	
18–30 years		222	(32)	270	(39)
31–60 years		410	(58)	398	(57)
More than 60 years		68	(10)	32	(4)
**Education**					
Illiterate		326	(47)	299	(43)
Literate		374	(53)	401	(57)
**Marital status**					
Unmarried		140	(20)	120	(17)
Married		560	(80)	580	(83)
**Place of residence**					
Rural		447	(64)	461	(66)
Urban		253	(36)	239	(34)
**Occupation**					
	Salaried (Government/Private)	139	(20)	162	(23)
	Daily wage labourer/ Agriculture	379	(54)	388	(55)
	Unemployed/housewife/student	182	(26)	150	(21)
**No. of household members**					
	≤ 5 members	470	(67)	453	(65)
	> 5 members	230	(33)	247	(35)

Of the interviewed respondents, 666 (95%) G2D/G1D, and 461(66%) G0D were of multi-bacillary type leprosy. The first symptom reported was reported as a skin patch with loss of sensation in 58% cases and 88% controls. Numbness in the hand or feet was noticed in 28% G2D/G1D; ulcers/blisters in the hand or feet were reported in 6% G2D/G1D. Symptoms suggestive of claw hand/foot drop/lagophthalmos was reported in 5% G2D/G1D. Non-skin lesions related to leprosy reaction symptoms was reported as first reported symptoms in 12% G2D/G1D and 9% G0D. Though various symptoms were reported by cases and controls, the chief trigger symptom that promoted the health seeking behaviour were skin patch with loss of sensation in 58% G2D/G1D and 88% G0D. Among G2D/G1D, numbness and reaction related symptoms contributed as a trigger symptom in 40% to seek health care from the providers. After noticing the first symptom, 58% G0D first consulted public health facility compared to 42% G2D/G1D. Among the remaining respondents, 42% G2D/G1D and 29% G0D sought care from qualified private practitioners i.e. Modern Medicine (MBBS & above) or Indian System of Medicine—Ayurveda Yoga, Unani, Siddha and Homeopathy (AYUSH) and 16% G2D/G1D and 13% G0D sought non-qualified practitioners (Practitioners with no medical degree) (**Table 2**).

**Table 2:** Disease related factors among G1D/G2D (cases) and G0D (controls) studied in the 5 states of India during 2014–2016.

Variables	G1D/G2D (n = 700)		G0D (n = 700)	
	n	(%)	n	(%)
**Leprosy type**				
MB	666	(95)	461	(66)
PB	34	(5)	239	(34)
First reported symptom*				
Skin patch with loss of sensation	406	(58)	616	(88)
Nodules	35	(5)	21	(3)
Non-Skin lesions (leprosy reaction related symptoms)	84	(12)	63	(9)
Numbness	196	(28)	-	
Ulcers	42	(6)	-	
Claw hand/foot drop/lagophthalmos	35	(5)	-	
**First health care provider consulted**				
Non-qualified practitioner	111	(16)	92	(13)
AYUSH private practitioner	67	(10)	52	(7)
Modern Medicine private practitioner	227	(32)	151	(22)
Public health system	295	(42)	405	(58)
**Distance to nearest public health facility**				
More than 5km	274	(39)	248	(35)
Less than or equal to 5km	426	(61)	452	(66)
**Delay**	Median (IQR)**		Median (IQR)**	
Patient delay in months	8 (2–24)		4 (1–12)	
HCP delay in months	2 (0–8)		1 (0–4)	
Total delay in months	14 (6–33.5)		6.2 (2.4–18)	
**Patient delay**				
More than 3 months	449	(64)	345	(49)
Less than or equal to 3 months	251	(36)	355	(51)
**HCP delay**				
More than 1 month	388	(55)	273	(39)
Less than or equal to 1 month	312	(45)	427	(61)

* Symptoms are not mutually exclusive

**Mann-Whitney test p<0.001

Data of delay in months was not normally distributed (using Kolmogorov-Smirnov test), hence median duration was used to present the delay instead of mean duration. The median patient delay was 8 months in G1D/G2D and 4 months in G0D; median HCP delay was 2 months in G1D/G2D and 1 month in G0D. The median total delay was 14 months in G1D/G2D and 6.2 months in G1D/G2D and about 61% G1D/G2D and 66% G1D/G2D reported nearest public health facility was less than 5 km (**Table 2**).

**Fig 3:**
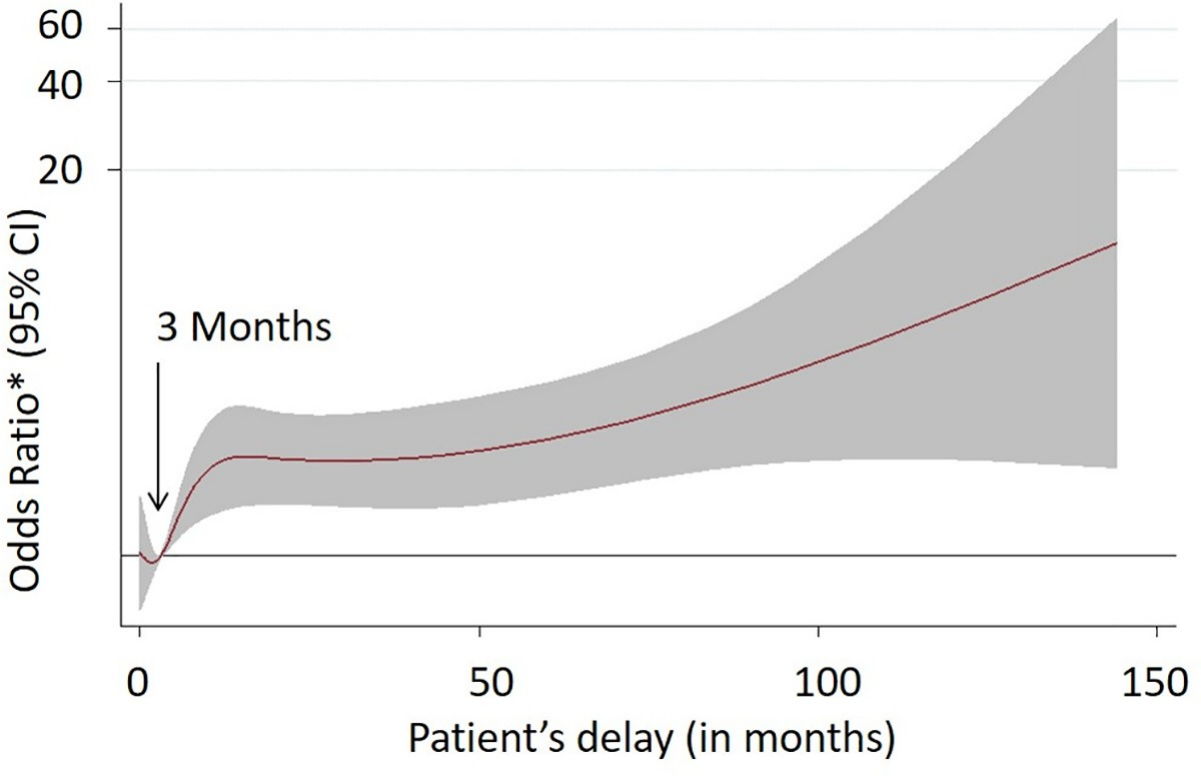
Non-linear relationship between patient delay and odds of G2D/G1D among adult leprosy cases at the time of diagnosis in five states of India during 2014–2016.

The non-linear relationship between patient delay and odds of G2D/G1D among adult leprosy cases at the time of diagnosis in five states of India implies three months patient delay as a significant risk factor for G2D/G1D among adult leprosy patients **(**Fig 3).

Risk factors for G2D/G1D among adult leprosy cases in 5 states of India, 2014–2016 is shown in Table 3. Univariate and multivariate logistic regression presented with odds ratio and 95% CI for the variables that predict the delay in leprosy diagnosis among adults with leprosy (**Table 3**). Multivariate logistic regression shows that the age of the respondent, leprosy type, occupation as a daily wage labourer, a patient delay of more than three months and HCP delay of more than one month were associated with risk of G2D/G1D at the time of diagnosis. Adult leprosy patients aged more than 60 years were twice more likely to present with G2D/G1D at the time of diagnosis compared with respondents aged less than 30 years (adj OR = 2.2, 95% CI: 1.3–3.6) and patients with MB type leprosy were nine times more likely to present G2D/G1D at the time of diagnosis compared to PB type leprosy (adj OR: 9.1, 95% CI: 6.2–13.3). Respondents who are daily wage labourer/ agriculture workers were 1.5 times more likely to present with G2D/G1D at the time of diagnosis compared to salaried (monthly income generating) person (adj OR = 1.5, 95% CI: 1.1–2.2).

**Table 3:** Risk factors for G2D/G1D among adult leprosy cases in 5 states of India, 2014–2016.

Variables	G2D/G1D (n = 700)		G0D (n = 700)		Crude OR (95% CI)	Adj OR (95% CI)
	n	(%)	n	(%)		
**Age at diagnosis**						
Less than 30 years	222	(32)	270	(39)	1.0	1.0
31–60 years	410	(58)	398	(57)	1.3(1.0–1.6)	1.2(0.9–1.6)
More than 60 years	68	(10)	32	(4)	2.6(1.6–4.1)	**2.2(1.3–3.6)**
**Occupation**						
Salaried (Government/Private)	139	(20)	162	(23)	1.0	1.0
Daily wage labourer/Agriculture	379	(54)	388	(55)	1.1(0.9–1.5)	**1.5(1.1–2.2)**
Unemployed/Housewife/Student	182	(26)	150	(21)	1.4(1.03–1.9)	1.2(0.9–1.7)
**Leprosy type**						
**MB**	666	(95)	461	(66)	10.1(7.0–14.8)	**9.1(6.2–13.3)**
PB	34	(5)	239	(34)	1.0	1.0
**Message related to leprosy/leprosy program**						
Not heard/seen/read	554	(79)	517	(74)	1.3(1.1–1.6)	1.1(0.8–1.4)
Heard/seen/read	146	(21)	183	(26)	1.0	1.0
**First health care provider consulted**						
Non-qualified practitioner	111	(16)	92	(13)	1.0	1.0
AYUSH Private Practitioner	67	(10)	52	(7)	1.1(0.7–1.7)	0.9(0.6–1.5)
Modern Medicine private practitioner	227	(32)	151	(22)	1.2(0.9–1.8)	1.2(0.8–1.8)
Public health system	295	(42)	405	(58)	0.6(0.4–0.8)	0.8(0.5–1.1)
**Patient delay**						
More than 3 months	449	(64)	345	(49)	1.8(1.5–2.3)	**1.6 (1.3–2.1)**
Less than or equal to 3 months	251	(36)	355	(51)	1.0	1.0
**Health care provider delay**						
More than 1 month	388	(55)	273	(39)	1.9(1.6–2.4)	**1.4(1.1–1.9)**
Less than or equal to 1 month	312	(45)	427	(61)	1.0	1.0

The adjusted odds ratio for respondents who preferred public health system against unqualified practitioner had a protective efficacy to present with G0D at the time of diagnosis (adj OR = 0.8, 95% CI: 0.5–1.1), but this finding was not statistically significant. When the patient delay was more than three months, the odds of G2D/G1D at the time of diagnosis was significantly higher (Adj OR = 1.6, 95% CI: 1.3–2.2) among respondents compared to those with patient’s delay of less than three months. This was statistically significant. When the HCP delay was more than one month the odds of G2D/G1D at the time of diagnosis was 1.4 times higher compared to those with less than one-month HCP delay (adj OR = 1.4, 95%CI: 1.1–1.9) and this was not statistically significant (**Table 3**). Notably, the majority of cases 554 (79%) and 517 (74%) G0D had not heard/seen/read message related to leprosy/leprosy program before their diagnosis.

A total of 652 out of 700 cases reported for not seeking any HCP after the notice of the first symptom. Ninety percent cases reported that they did not know the symptom they experienced was due to Leprosy and felt that the symptom would disappear by itself (**Table 4**).

**Table 4:** Reason for not seeking any health care provider after noticing the first symptom among adult leprosy cases with G2D/G1D.

Reason for not seeking any health care provider after noticing symptom*	Cases n = 652 (%)
Thought it was a not disease and would disappear by itself	585 (90)
No money to seek treatment	144 (22)
Due to family commitments	81 (12)
Health facility is far away	51 (8)

*Multiple responses

## Discussion

In this study, age and gender of the G1D/G2D (cases) and G0D (controls) were comparable. The main risk factors for disability among adult leprosy cases with G2D/G1D were multi-bacillary leprosy, patient delay of more than 3 months, HCP delay of more than 1 month, daily wage laborer and age > 60 years were the significant risk factors for presenting with G2D/G1D at the time of diagnosis among the newly diagnosed leprosy patients. In this study, multi-bacillary leprosy patients had nine times higher odds of G2D/G1D compared to pauci-bacillary leprosy type and this finding was similar to the retrospective, descriptive and exploratory investigation of 19,283 patients with leprosy in Brazil [19].

Detailed analysis of various factors interplaying to affect the health-seeking behaviour and timely diagnosis showed that they could be categorised into either patient-related or health care provider-related factors. The former being the major factor contributor to the delay in both G2D/G1D and G0D. Nicholls et al and colleagues (2003) found in a study that the delay in diagnosis among individuals with G2D was double that for individuals with no disability, which is in line with the current study [20]. This study showed that the median total delay was 14 months among cases with disabilities. The evidence from the BANDS study (2003) suggests that a threshold of no more than six months might be used to define early and late presentation [20]. However, in this study patient delay of even more than three months emerged to be a significant risk factor much higher when compared to by BANDS study. Patient delay contributed two-thirds of the total delay in diagnosis. A patient delay of more than 3 months should be considered as an indicator for poor IEC penetration in the community.

In this study, cases reported that skin lesion viz., increase in the number of skin patches, raised patches, sensory impairment (numbness in hand/feet) triggered the respondents to seek a health care provider. Nicholls et al. (2003) in their study found that signs of leprosy reaction to be a trigger factor for early presentation [20]. In a study by Mendiratta et al. (2006) paraesthesia and numbness were the presenting complaints in 63% new leprosy patients, and motor deficit (paresis) in 35% patients; deformities (claw hand, foot drop, trophic changes) were seen in 50% patients [21]. The study by Shumet et al. (2015) in Ethiopia reported that those who presented with sensory loss were also more likely to have a disability at the time of diagnosis [22]. A study by Samraj et al (2012) reported that 61% of patients of their study participants had a disability at first presentation [23]. Since leprosy may present with myriad symptoms, it is necessary that Information, Education and Communication (IEC) activities should now place emphasis on other symptoms as well, along with an improved focus on hidden patches.

Ninety percent of the study participants reported that they were not aware of the leprosy symptoms. Ignorance of illness or waiting for the disappearance of the symptom by itself was the major reason for not seeking help in the majority of the cases. A study at Columbia also reported that 90% of the study participants not sought any HCP after noticing the first symptom [24]. Henry and colleagues reported that participants who thought their symptoms were not serious had a threefold greater chance of waiting longer before consulting than those who did. [5].

HCP delay of more than a month implies the lacunae of the health system to diagnose leprosy at an early stage. Patient’s disability could have worsened during his/her health seeking pathway or it took relatively long time for HCPs to diagnose leprosy among patients with G2D/G1D (cases) than among G0D (controls). This could be due to their inability to suspect and diagnose leprosy in time. Another possible reason is that leprosy is a disease of stigma and most of the time the doctors refer the patients to a higher centre for leprosy diagnosis by misinforming the patients as a simple skin problem and not by naming as leprosy or not informing the consequences of leprosy [25]. This could have made patients to give less importance for the referral leading to late diagnosis. Despite all these factors, access to the health facility was not reported as an issue in this study.

With the onset of symptoms, patients initially practiced self-medication, visited local healers, or chemists /pharmacies. Meanwhile, they sought some health care provider. These different actions varied across G2D/G1D and G0D. The majority of G2D/G1D first sought care from private practitioners (unqualified practitioner/ AYUSH practitioner or modern medicine system) while G0D sought from the public health facility. Within the private sector, qualified private practitioners were consulted by most cases. This finding has to be interpreted in the following ways. Firstly, some of the interviewed patients reported of being prescribed investigations like X-rays, CT, MRI, miscellaneous drugs and treatment suggesting that leprosy was not suspected at first sight. However, at least some of these may have been appropriate responses to concurrent infections or other conditions not related to leprosy and to eliminate other skin conditions as a legitimate approach. Hence it would be inappropriate to label all these as occasions as a failure by HCPs to diagnose leprosy. Secondly, some individuals claimed that they did not know their doctor’s diagnosis, while others admitted they had not complied with referrals. Thirdly, many individuals did not stay consistent with follow-up, tending to move on from one doctor to another, either seeking a second opinion or an alternative acceptable diagnosis. Ignorance of illness or waiting for the disappearance of the symptom was the reason for not seeking help in the majority of the cases.

But the results of this study indicate that HCP delay also have played a role in risk of developing disability among adult leprosy cases at diagnosis. The majority of cases (58%) of the study patients had first approached the private sector for healthcare as against public health sector due to less waiting time/suitable out-patient timings and lesser stigma [26].

A study in West Bengal reported a mean total delay of 19.6 months [27]. It was lesser than the mean total delay of 37 months among G2D cases which was reported by a study conducted in Purulia [28]. A study in a South Indian at a tertiary care hospital revealed that mean patient delay was 7.9 months [29]. Internationally, Bekri and colleagues in 1998 reported a total delay of 26 months among G2D/G1D and 12 months among G0D [3]. Souza and colleagues in 2003 reported that 45% of the study patients had a leprosy diagnosis within a year of notice of symptoms [30]. In Nepal 50% of the study participants delayed more than 18 months from the notice of first symptom to diagnosis [4]. Even after the declaration of elimination of leprosy as a public health problem in the year 2005, the duration delay in our study is similar to the findings from the studies mentioned above.

### Limitations of the study

This study was conducted in five states of India selected based on the operational feasibility and leprosy burden during 2012–13 and this could have resulted in selection bias. The study findings might be applicable to southern, eastern, western and northern region of India, however, it should be interpreted with caution while applying the study findings to central and north-eastern part of India due to the non-inclusion of states from these regions.

We attempted to adjust for confounding based on leprosy type (PB/MB) during analysis but the proportion of PB among the cases was only 5% of the overall sample so we could not do a stratified analysis for PB/MB. Additionally, any possible matching for leprosy type (PB/ MB) at subject recruitment phase would not have allowed us to analyse the association of this factor (PB/MB) with disability. Further, the study design is a case control design and has an inbuilt reporting bias due to the retrospective nature of data collection but the data collection was carried with as much caution to reduce the recall bias. Further. the study findings did not find a significant difference in the leprosy/program knowledge among G0D (26%) as compared to G2D/G1D (21%) and this is possibly due to small sample size. The matching ratio of cases and controls was 1:1 in this study, but we could not increase the controls due to operational feasibility during the conduct of the study.

### Conclusions

Our study found that the delay in diagnosis is still a major challenge of leprosy program in India. Both patient delay (> 3 months) and healthcare provider delay (>1month) have been significant risk factors for disability among adult leprosy cases. In this study, a large proportion of cases with disability ignored the initial symptoms as they thought the symptoms would disappear by itself.

To reduce the patient-related delay, the program should lay greater emphasis on raising awareness of the community focusing on key messages like symptoms, the disability consequence of late detection, availability of free treatment, availability of leprosy care in public health facility. Additionally, program should put more emphasis on early case detection campaigns like active survey.

On the other hand, efforts should be made to periodically engage and capacitate all HCPs (all types of private health care providers along with the public health staff) to have a high index of suspicion of leprosy and facilitate effective referrals.

To further understand the specific local factors associated with the patient delay and HCP delay, we suggest that disability audit should be conducted on a routine basis for each leprosy case diagnosed with disability.

## Supporting information

S1 Checklist Strobe checklist. (DOCX)

S1 File Data set. (XLS)
