# Supplementary material for: Risk of disability among adult leprosy cases and determinants of delay in diagnosis in five states of India: A case-control study
Source: PLoS Negl Trop Dis. 2019 Jun 27;13(6):e0007495. doi: 10.1371/journal.pntd.0007495 (PMC6619834; doi:10.1371/journal.pntd.0007495)
Supplement: S1 Checklist — (DOCX) [file pntd.0007495.s001.docx]

S2: STROBE Statement—checklist of items that should be included in reports of observational studies

|  | | Item No | Recommendation | Line No |
| --- | --- | --- | --- | --- |
| **Title and abstract** | | 1 | (*a*) Indicate the study’s design with a commonly used term in the title or the abstract | Abstract – Methods, 1^st^ paragraph |
|  |  |  | (*b*) Provide in the abstract an informative and balanced summary of what was done and what was found | Abstract – Introduction, methods, results and conclusion |
| Introduction | | | |  |
| Background/rationale | | 2 | Explain the scientific background and rationale for the investigation being reported | Introduction paragraph – 1, 2 and 3 |
| Objectives | | 3 | State specific objectives, including any prespecified hypotheses | Introduction – 3^rd^ paragraph |
| Methods | | | |  |
| Study design | | 4 | Present key elements of study design early in the paper | Methods – 2^nd^ paragraph |
| Setting | | 5 | Describe the setting, locations, and relevant dates, including periods of recruitment, exposure, follow-up, and data collection | Methods – 2, 3 and 4^th^ paragraph |
| Participants | | 6 | (*a*) *Case-control study*—Give the eligibility criteria, and the sources and methods of case ascertainment and control selection. Give the rationale for the choice of cases and controls | Methods – 5^th^ Paragraph |
|  |  |  |  |  |
| Variables | | 7 | Clearly define all outcomes, exposures, predictors, potential confounders, and effect modifiers. Give diagnostic criteria, if applicable | Methods – 8^th^ Paragraph |
| Data sources/ measurement | | 8* | For each variable of interest, give sources of data and details of methods of assessment (measurement). Describe comparability of assessment methods if there is more than one group | Methods – 5^th^ Paragraph |
| Bias | | 9 | Describe any efforts to address potential sources of bias | Methods – 6^th^ Paragraph |
| Study size | | 10 | Explain how the study size was arrived at | Methods – 8^th^ Paragraph |
| Quantitative variables | | 11 | Explain how quantitative variables were handled in the analyses. If applicable, describe which groupings were chosen and why | Methods – 6^th^ Paragraph |
| Statistical methods | | 12 | (*a*) Describe all statistical methods, including those used to control for confounding | Methods – 8^th^ Paragraph |
|  |  |  | (*b*) Describe any methods used to examine subgroups and interactions |  |
|  |  |  | (*c*) Explain how missing data were addressed |  |
|  |  |  | (*d*) *Case-control study*—If applicable, explain how matching of cases and controls was addressed |  |
|  |  |  | (*e*) Describe any sensitivity analyses |  |
| Results | | | |  |
| Participants | 13* | (a) Report numbers of individuals at each stage of study—eg numbers potentially eligible, examined for eligibility, confirmed eligible, included in the study, completing follow-up, and analysed | | Results – 1^st^ paragraph |
|  |  | (b) Give reasons for non-participation at each stage | | NA |
|  |  | (c) Consider use of a flow diagram | | - |
| Descriptive data | 14* | (a) Give characteristics of study participants (eg demographic, clinical, social) and information on exposures and potential confounders | | Results – 1^st^ paragraph and table 1 |
|  |  | (b) Indicate number of participants with missing data for each variable of interest | | NA |
|  |  |  | |  |
| Outcome data | 15* |  | |  |
|  |  | *Case-control study—*Report numbers in each exposure category, or summary measures of exposure | | Results – 1^st^ paragraph and table 1 |
|  |  |  | |  |
| Main results | 16 | (*a*) Give unadjusted estimates and, if applicable, confounder-adjusted estimates and their precision (eg, 95% confidence interval). Make clear which confounders were adjusted for and why they were included | | Table 2 and 3, Figure 2 |
|  |  | (*b*) Report category boundaries when continuous variables were categorized | | Table 2 and 3, Figure 2 |
|  |  |  | |  |
| Other analyses | 17 | Report other analyses done—eg analyses of subgroups and interactions, and sensitivity analyses | | Table 2, 3 and 4 2 |
| Discussion | | | |  |
| Key results | 18 | Summarise key results with reference to study objectives | | Discussion paragraph 1-7 |
| Limitations | 19 | Discuss limitations of the study, taking into account sources of potential bias or imprecision. Discuss both direction and magnitude of any potential bias | | Limitation paragraph 1 |
| Interpretation | 20 | Give a cautious overall interpretation of results considering objectives, limitations, multiplicity of analyses, results from similar studies, and other relevant evidence | | Limitation paragraph 1 and conclusion paragraph 1 |
| Generalisability | 21 | Discuss the generalisability (external validity) of the study results | | Limitation paragraph 1 and conclusion paragraph 1 |
| Other information | | | |  |
| Funding | 22 | Give the source of funding and the role of the funders for the present study and, if applicable, for the original study on which the present article is based | | Acknowledgment 2^nd^ paragraph |
